# Successful treatment of resistant plantar ulcerative lichen planus with tofacitinib: A case report and comprehensive review of the literature

**DOI:** 10.1002/ccr3.8066

**Published:** 2023-10-17

**Authors:** Afsaneh Sadeghzadeh Bazargan, Alireza Jafarzadeh, Niloufar Najar Nobari

**Affiliations:** ^1^ Department of Dermatology, Rasool Akram Medical Complex Clinical Research Development Center (RCRDC), School of Medicine Iran University of Medical Sciences (IUMS) Tehran Iran; ^2^ Skin and Stem Cell Research Center Tehran University of Medical Sciences Tehran Iran

**Keywords:** case report, dermatology, erosive, jak inhibitor, lichen planus, lichen planus, plantar erosion, plantar ulcer, tofacitinib, ulcerative lichen planus

## Abstract

**Key Clinical Message:**

Ulcerative lichen planus, a challenging variant of lichen planus, has limited response to traditional treatments. Tofacitinib, a JAK–STAT pathway inhibitor, shows promise in effectively treating these lesions.

**Abstract:**

Lichen planus is a mucocutaneous disease that can cause various manifestations, such as itchy erythematous papules, cicatricial alopecia, erosion, and mucocutaneous ulcers. One uncommon manifestation of this disease is the occurrence of erosion and skin ulcers in the soles of the feet, which can cause many problems for patients. Tofacitinib, a Janus Kinase (JAK) inhibitor drug, has found a special place in the field of inflammatory diseases, especially inflammatory skin diseases. In this regard, studies on the effective role of this drug in the treatment of certain forms of lichen planus, including lichen planopilaris, and erosive lichen planus have been performed. In upcoming study, we introduce a 52‐year‐old woman with lichen planus who complained of ulcerative lesions on the sole of her foot, for whom a diagnosis of plantar ulcerative lichen planus was proposed. After the patient did not respond therapeutically to intralesional triamcinolone acetonide injection, as well as methotrexate and cyclosporine tablets, significant improvement was finally achieved with a 5 mg twice daily dose of tofacitinib. In the following, we will comprehensively review previous articles on the role of tofacitinib in the treatment of lichen planus lesions, as well as the proposed treatment options for erosive and ulcerative lichen planus lesions specifically located on the sole of the foot. Despite limited reports of the successful treatment of mucosal erosive lesions in the oral, esophageal, genital, and ocular mucosa areas with tofacitinib, no previous study has reported the successful treatment of ulcerative lichen planus lesions of the plantar area with tofacitinib. While reporting this case, we recommend considering tofacitinib as a treatment option for plantar ulcerative lichen planus. To confirm its effectiveness, it is necessary to conduct more extensive studies with a larger sample size.

## Graphical abstract

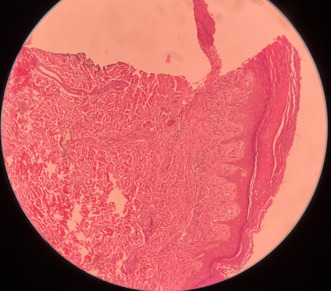

## 1 INTRODUCTION

Lichen Planus is a skin disease that can also affect the mucous membranes and nails.^1^, ^2^ The skin manifestations of this disease are multiple purple papules often accompanied by itching.^3^ The mucosal involvement of this disease is known as mucosal erosions that often involve the oral mucosa and genital area.^4^

One of the rare types of this skin disease is called ulcerative lichen planus, which presents as chronic ulcers resistant to treatment, associated with pain and many disabilities for patients.^5^, ^6^ The involvement of the soles of the feet is one of the most significant areas affected by ulcerative lichen planus. Despite several treatment options having been studied in the past, treating plantar ulcerative lichen planus remains a challenge for dermatologists.^4^, ^5^, ^6^, ^7^, ^8^

Tofacitinib, a Janus Kinase inhibitor, has a special place in the treatment of inflammatory diseases in medicine.^9^ Recently, studies have shown its effectiveness in treating various skin diseases such as psoriasis, alopecia areata, vitiligo, and atopic dermatitis.^10^ In recent studies, the effectiveness of this drug in treating certain types of lichen planus has been mentioned, including lichen planopilaris and erosions of the oral mucosa.^9^, ^11^

In this article, we presented a case of plantar ulcerative lichen planus that was initially resistant to treatment but exhibited a remarkable response upon being treated with oral tofacitinib. In addition, we conducted a comprehensive review of literature on the efficacy of tofacitinib in treating various types of lichen planus lesions, while also discussing other proposed treatments for plantar ulcerative lichen planus.

## 2 CASE PRESENTATION

**FIGURE 1:**
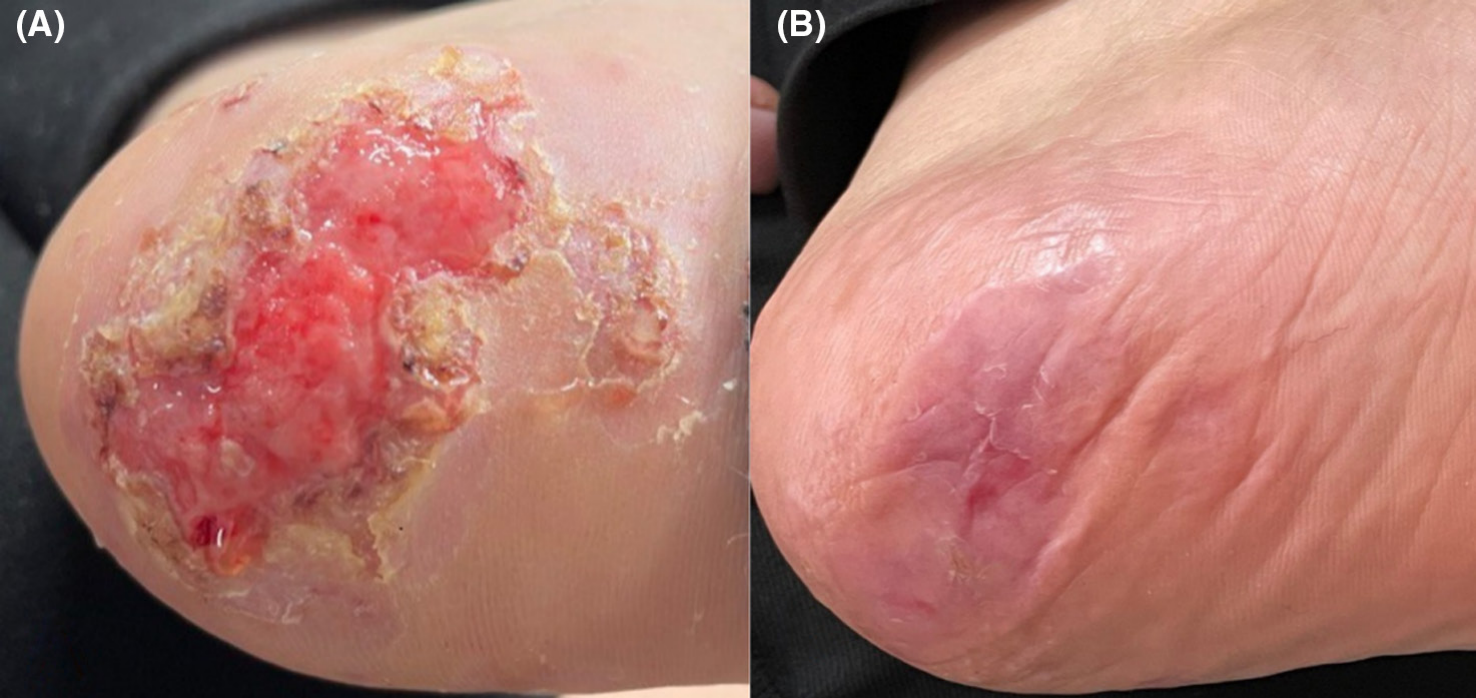
Improvement of plantar ulcerative lichen planus after receiving tofacitinib. (A) A patient with lichen planus presented with a treatment‐resistant ulcer on the sole of their foot. (B) There was an improvement in the ulcer after the patient received one month of tofacitinib treatment.

A 52‐year‐old woman with a known case of lichen planus was referred to our clinic with a large ulcerative lesion in the plantar area. Her lichen planus disease started 9 years ago with skin involvement and erosion of the oral mucosa. The patient was treated with cyclosporine tablets 100 mg three times a day and triamcinolone acetonide injection with a concentration of 5 mg/mL in the mucosal erosion site, which resulted in improvement of her mucosa and skin condition during the 3‐year treatment period. No other diseases were mentioned in the medical history taken from her, and no drug treatments were mentioned except for the one prescribed for the treatment of lichen planus. Three years ago, the patient developed erythema in the sole area, which gradually progressed over 2 years and eventually led to erosion and ulceration (Figure 1).

**FIGURE 2:**
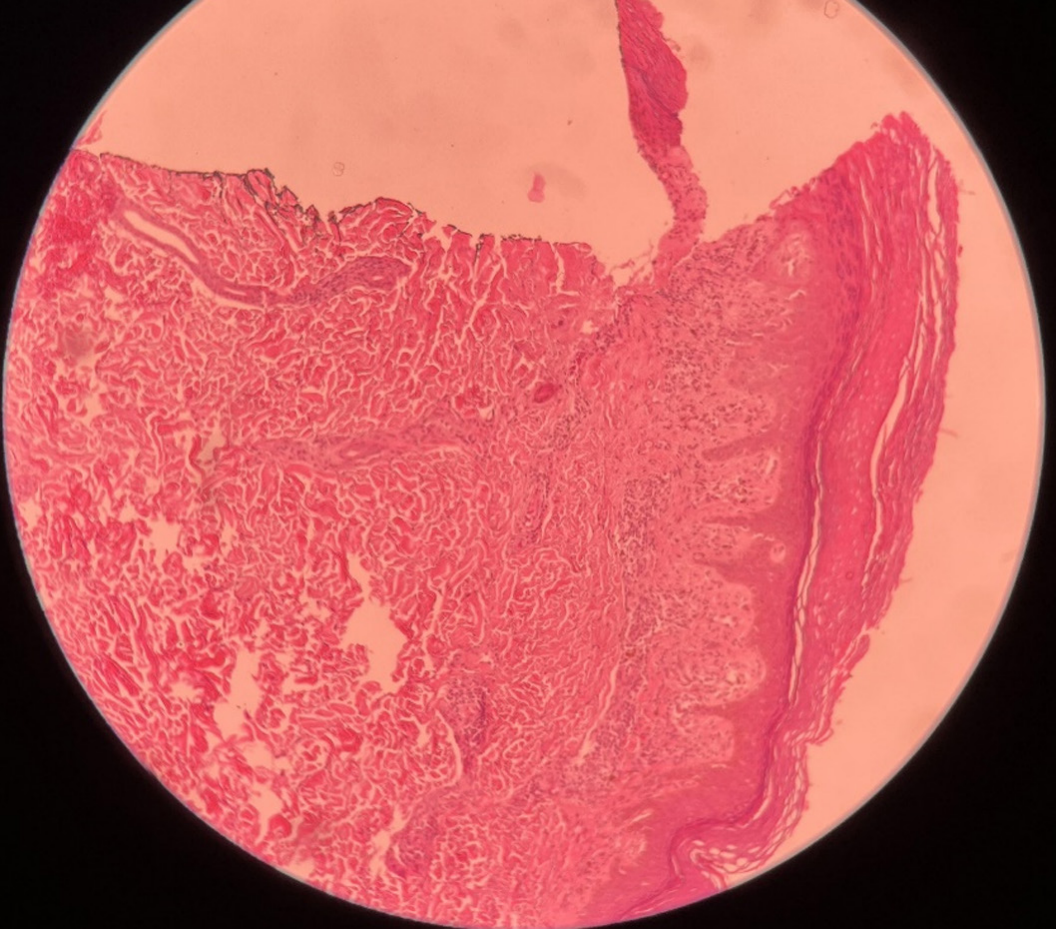
Histopathological appearance of lichen planus lesion. Hyperkeratosis, acanthosis, hypergranulosis, basal layer degeneration, and lymphocyte bandlike infiltration are evident.

A biopsy sample was taken from the lesion, and the pathologist reported findings consistent with lichen planus, including hyperkeratosis, irregular acanthosis, hypergranulosis, basal layer degeneration, band‐like lymphocytic infiltration, civatte body, and melanin incontinence. Furthermore, no evidence of atypia or SCC (squamous cell carcinoma) was detected (Figure 2). Despite the treatment with cyclosporine tablets (100 mg, three times a day), no satisfactory clinical improvement was observed in the lesion. Additionally, the patient experienced an increase in blood pressure following the use of the drug. As a result, the medication was discontinued, and the patient was referred to our clinic for further evaluation and treatment.

After obtaining a detailed medical history, a clinical examination was performed on the patient. The examination revealed an ulcer in the sole of the foot with an irregular and crusted border. There were no signs of infection, and the lesion was exuding. No skin or muocutaneous lesions were observed in other areas. Upon reviewing the pathology report and considering the patient's refusal to undergo further sampling, a diagnosis of ulcerative lichen planus was proposed.

Routine blood tests were without pathological findings. She was prescribed methotrexate tablets at a dose of 15 mg per week and triamcinolone acetonide injection with a concentration of 5 mg/mL at monthly intervals as part of her treatment plan. After 8 months from the start of the treatment, there was partial recovery in the lesions, but the patient's ulcer had not completely healed. Therefore, after discontinuing methotrexate, a re‐examination of blood tests was requested, including a complete blood count, liver function test, kidney function test, lipid profile test, PPD, HCV Ab, HBsAg, HBsAb, and HIV Ab. All the results came back normal. Following this, a prescription for tofacitinib 5 mg tablets twice a day was provided. The duration between discontinuing methotrexate and starting tofacitinib was 1 week. After completing a 1‐month course of the drug, the patient's ulcer completely healed, and no side effects were reported by the patient (Figure 1). The patient underwent a 3‐month treatment with tofacitinib, taking 5 mg twice a day. Throughout this period, the patient solely relied on tofacitinib and did not make use of any other oral or topical medications. Once the tofacitinib treatment was completed, it was discontinued, and instead, tacrolimus ointment was applied twice a week. Interestingly, within just one month of starting the tacrolimus ointment treatment, no recurrence of lesions was observed. No side effects were observed during the entire treatment.

## 3 DISCUSSION

Plantar ulcerative lichen planus is a rare form of lichen planus that causes disability and numerous problems for patients.^6^ Various treatments have been proposed for this type of disease, which have been associated with varying degrees of recovery.^5^, ^6^, ^7^, ^8^ In this article, we present the case of a 52‐year‐old woman who had a large ulcer on the sole of her foot that did not heal completely with previous treatments, including cyclosporine, methotrexate, and triamcinolone acetonide intralesional injection. She was then prescribed tofacitinib 5 mg tablets to be taken twice a day, and as a result, the lesion has now completely healed.

Janus Kinases play an essential role in the transmission of gamma interferon signals, which are important mediators in the pathogenesis of lichen planus. Based on this, the drug tofacitinib, which inhibits all types of Janus kinases, should theoretically be effective in the treatment of lichen planus.^2^ In a retrospective review study published by Plante et al. in 2020, nine previous studies were examined in which lichen planopilaris patients responded to oral and topical treatments with tofacitinib. Among the studies reviewed, six studies investigated oral tofacitinib, and in most cases, a daily dose of 10 mg was prescribed.^9^

It is worth mentioning that another study conducted by Damsky et al. in 2020 reported significant improvement in three patients with treatment‐resistant erosive lichen planus. These patients had not responded to treatment with cyclosporine, methotrexate, acitretin, prednisone, and mycophenolate mofetil, but showed a dramatic response to treatment with tofacitinib at a dose of 5 mg twice a day.^11^

Comparing the above study with our study, it is worth noting that our study involved the plantar surface of the foot, while the other study involved areas such as oral, penile, ocular, and vaginal mucous membranes in three patients. The dose used in our study was similar to the other study, and complete or near‐complete treatment responses were seen in both studies. It is also worth mentioning that the lesions in both studies were resistant to previous treatments such as cyclosporine and methotrexate.

In a case report study conducted by Kozlov et al. in 2023, a case of severe esophageal lichen planus recovery after treatment with tofacitinib was reported. The study introduced an 89‐year‐old woman with lichen planus involving the skin, genital mucosa, mouth, and esophagus. The patient's esophageal lesions were resistant to treatment with cyclosporine. While the patient did not respond well to the initial treatment with tofacitinib at a dose of 5 mg daily, her dysphagia and weight loss improved after receiving a dose of 5 mg twice a day.^1^ The results of this study were consistent with our study, where both studies showed improvement of the lesion and absence of side effects and recurrence with a daily dose of 10 mg of tofacitinib. An important point in this study is the necessity of receiving the appropriate dose of the drug to control the lesions, as the lesions did not respond to a lower dose of the drug (5 mg daily). In this regard, the study by Kooybaran et al. introduced a 77‐year‐old female patient who complained of erosion of the oral mucosa and was diagnosed with esophageal lichen planus. The patient showed a positive response to treatment with tofacitinib.^12^

In a study conducted by Seiringer et al. in 2020, they reported a case of a 51‐year‐old patient with hypertrophic lichen planus who responded positively to treatment with tofacitinib at a dose of 5 mg twice a day. Although this patient had a different type of lichen planus compared to our patient, it highlights the effectiveness of tofacitinib in treating various types of lichen planus.^13^

A case report study by Kilic et al. (2017) documented successful treatment for erosive lichen planus with cyclosporine tablets in a 65‐year‐old woman with an erosive plaque on the sole of her foot. After 5 months of treatment at a dosage of 3 mg/kg per day, the patient showed significant improvement.^4^ However, the outcome of this study differs from that of your study, as the patient's lesion in Kilic et al.'s study was erosive, while yours was ulcerative.

This difference in lesion type may explain the varying results observed with the use of cyclosporine mist. It is possible that cyclosporine mist could be effective in treating initial forms of erosive lichen planus lesions in the soles of the feet, but its effectiveness could decrease as the lesion progresses to the ulcerative form. However, further comprehensive studies with appropriate sample sizes would be necessary to confirm this hypothesis.

In the Salavastru study conducted in 2010, a significant improvement in ulcerative lichen planus lesions on the soles of a 77‐year‐old woman was noted after 4 weeks of treatment with tacrolimus ointment 0.1% twice a day.^8^ However, the use of tacrolimus ointment in ulcerative lesions presents a challenge of systemic drug absorption, and caution should be taken in this regard.

Similarly, in the Kandula study conducted in 2018, a 56‐year‐old woman presented with a painful ulcer on the metatarsal and plantar level of the big toe, which responded dramatically to treatment with prednisone tablets 40 mg daily in combination with clobetasol ointment 0.05% twice a day for 2 weeks.^6^ However, the use of prednisone, particularly in older individuals, can have serious side effects.

In addition to the drug treatments discussed in the reviewed studies, surgical methods have also been reported to improve erosive and ulcerative lesions of the soles of the feet in lichen planus disease. For instance, Miotti et al. in 2020 reported successful treatment of erosive lichen planus in the plantar area with autologous micrografts and methotrexate tablets. The study involved a 65‐year‐old woman with a 6‐year history of foot sole ulceration caused by erosive lichen planus, who responded well to treatment with 15 mg of methotrexate tablets per week and autologous skin grafting from the thigh area.^7^ However, this study differs from ours since it combines drug therapy with surgery in treating ulcerative lesions of lichen planus. It's worth noting that using invasive and surgical methods in lesion treatment may not be acceptable to many patients.

If the effectiveness of these treatments is confirmed in wider studies, each patient's benefit and harm from each treatment method should be calculated separately. The summary of studies on erosive and ulcerative lichen planus in the sole area is presented in Table 1.

**TABLE 1:** Comparison of studies conducted on erosive and ulcerative lichen planus in the sole area from 2010 to 2023.

Variables	Our study	Kandula study^6^	Kilic study^4^	Salavastru study^8^	Miotti study^7^	Goucha study^5^
Gender	Female	Male	female	Female	Female	Female
Age (years)	52	56	65	77	65	38
Comorbidities	None	Tongue ulcer	Hashimoto's thyroiditis/History of cholecystectomy and hysterectomy	Erosion of the oral mucosa/Skin papules of lichen planus	Type 2 diabetes mellitus/hypertension/dyslipidemia/obesity/chronic renal failure/anemia/erosive gastritis	None
Treatment	Tofacitinib 5 mg tablet twice daily p.o.	Oral prednisone 40 mg once daily and clobetasol 0.5% twice daily	Cyclosporine tablet with a dose of 3 mg/kg/day	Tacrolimus 0.1% ointment	methotrexate 15 mg tablet once a week and Autologous micrografts	Oral prednisone 90 mg/day
The time between starting the drug and complete recovery	1 month	2 weeks	5 month	4 weeks	2 weeks	3 weeks

## 4 CONCLUSION

In this study, we report a case of plantar ulcerative lichen planus that showed a remarkable therapeutic response to treatment with tofacitinib tablets. We have introduced a suitable treatment option for these lesions and added another application to the set of tofacitinib's applications in treating skin diseases. It is recommended to conduct more extensive studies with a larger sample size in the future to confirm this therapeutic application.

## AUTHOR CONTRIBUTIONS

**Afsaneh Sadeghzadeh Bazargan:** Writing – original draft. **Alireza Jafarzadeh:** Writing – original draft. **Niloufar Najar Nobari:** Writing – review and editing.

## FUNDING INFORMATION

None.

## ETHICS STATEMENT

The researchers were committed and adhered to the principles of the Helsinki Convention and the Ethics Committee of the Iran University of Medical Sciences in all stages.

## CONSENT

After providing the necessary explanations, written informed consent was obtained from the patient regarding the submission of their clinical condition to medical journals. Additionally, the patient has been assured that their name and personal details will be kept confidential by the authors.

## TRANSPARENCY DECLARATION

Authors declare that the manuscript is an honest, accurate, and transparent. No important aspect of the study is omitted.

## Data Availability

All data produced in the present study are available upon reasonable request to the authors.
